# Supplementary material for: Harnessing whole human liver ex situ normothermic perfusion for preclinical AAV vector evaluation
Source: Nat Commun. 2024 Mar 14;15:1876. doi: 10.1038/s41467-024-46194-y (PMC10940703; doi:10.1038/s41467-024-46194-y)
Supplement: Supplementary file 1 — Supplementary Information [file 41467_2024_46194_MOESM1_ESM.pdf]

Supplementary Table 1. AAV vectors used in the study. AAV8 and the AAV8 variants belong to Clade E. AAV9 belongs to Clade F. AAV-hu.Lvr06, FT01 and FT11 are highly homologous to AAV2 (Clade B). AAV-LK03, AAV-SEQ3, and AAV-LK03-REDH are highly homologous to AAV3b (Clade C). AAV-SYD11 and AAV-SYD12 are highly homologous to AAV7 (Clade D). From the chosen serotypes, only AAV-SEQ3 and AAV-LK03 are known to bind strongly to Heparan Sulfate Proteoglycans (HSPG). AAV-LK03-REDH is an HSPG de-targeted variant of AAV-LK03, and AAV-hu.Lvr06 is a naturally-occurring HSPG de-targeted variant of AAV2. All of the natural (wild-type) serotypes rely on the AAV-Receptor (AAVR), for successful cellular entry and subsequent infection.[1] This dependency is also expected to be true for all bioengineered serotypes, given their genetic lineage from natural strains. The selection of wild-type serotypes and AAV-SEQ3 is based on their relevance and application in clinical settings.

AAV variants	Origin	Method	Composition	Reference
AAV5	Nature - Human	Isolation	Wild-type	6324476
AAV8	Nature - NHP	Isolation	Wild-type	PMC129358
AAV9	Nature - Human	Isolation	Wild-type	PMC416542
AAV-hu.Lvr06	Nature - Human	Isolation	Wild-type – AAV2-like	32908003
AAV-LK03	Bioengineered	Directed Evolution - hFRG	Shuffled – AAV3b-like	24390344
AAV-SEQ3	Bioengineered	Empirical domain swap	Domain Swap – AAV3b-like	WO/2016/181123
AAV-FT01	Bioengineered	Directed Evolution - hFRG	Peptide Displayed on AAV2	35297686
AAV-FT11	Bioengineered	Directed Evolution - hFRG	Peptide Displayed on AAV2	35297686
AAV-SYD11	Bioengineered	Directed Evolution - hFRG	Shuffled – AAV7-like	34977275
AAV-SYD12	Bioengineered	Directed Evolution - hFRG	Shuffled – AAV7-like	34977275
LK03-REDH	Bioengineered	Rational Design	R594E+D598H	36700121
AAV8-Swap03	Bioengineered	Domain Swap - hFRG	AAV8 with VR-VI to VIII from AAV7	34977275
AAV8-Swap05	Bioengineered	Domain Swap - hFRG	AAV8 with VRI from AAV2 + VR-VI to VIII from AAV7	34977275
AAV8-Swap06	Bioengineered	Domain Swap - hFRG	AAV8 with VR-IV and V from AAV10 + VRVI-VIII AAV7	34977275

Supplementary Table 2. Donor Demographics. DBD – Donor after brain death. DCDD – Donation after circulatory determination of death.

Donor ID	Donor Age	Donor Sex	Graft Weight (g)	Donor Type
1	78	F	1615	DBD
2	56	M	2188	DCDD

Supplementary Table 3. Barcode Sequences.

Barcode	Sequence (5' to 3')
BC 002	GATAGGGAAGCAAAGCAAACTAAACTGAACGGAACGAAGGGGAA
BC 003	TTAACTGTAGCGTGCGGAACGACACTTTCCGACCCTGACCTGCA
BC 005	GGTCGCTTGGTGTAGGTACGGTCACGACAGTGAACCTCGGCATC
BC 009	TGTAGGGAGGTGTACTGTCCCGAACCGTAGGGAAGTGCGGGAAC
BC 010	TTCGCTTAGCTATGCCTCAGTTAACGACCCTGCACTTCAGCACC
BC 011	TGAACCTTAGCGAGCTACCGTATGGCATGGGGAACCATCCGACC
BC 012	TTAACTTAAGCGCACCACAGGATAGCAAAGTTCACCAAACTTAA
BC 013	TACACGGTCCTTTGGGACACTGCGGTACAGCAAAGCGTCCTACC
BC 014	TTTGGTGTGCGTCCCCAAACCAAACTAACCGGCGCCTTACGACA
BC 015	CTAACTGAAGTACCCGACGCCGCCGGGAGCTTTACTGAACCTAA
BC 018	CGAAGCTAGGTGCACCAAACCTTGCGTAACCACGCTGTGGCAAA
BC 020	TACACCTAAGTGACCTTTCCCTAAGGTCAGCGACGTTTGGTTAA
BC 022	CTTCGTTAAGCTCCGCGACCCTAACTGCACGGAACGATAGGAAA
BC 023	GTAGGGAAGCGATGCCTACCGGAGGGAAGGCGCACCAACGTTCA
BC 024	TGTACCAAACGGAAGGGAATCTTGCCACACTACGCCAACCGGTG

Supplementary Table 4. Overview of AAV Model Characteristics and Transduction Efficacy Observed in the Study.

	Model Features				
**Model**	**Human Origin**	**Periferal Organs**	**Neutralizing Antibodies**	**Adaptive Immunity**	**Liver Immune Resident Cells**
Liver Explant	Yes	No	No	No	Yes
Liver Explant + Neutralizing Antibodies	Yes	No	Yes	No	Yes
HuH-7	Yes	No	No	No	No
Naïve FRG	No	Yes (murine)	No	No	Low/No
Humanized FRG Low	Xenografted	Yes (murine)	No	No	Low/No
Humanized FRG Mid	Xenografted	Yes (murine)	No	No	Low/No
Humanized FRG High	Xenografted	Yes (murine)	No	No	Low/No
Humanized FRG High (2 months)	Xenografted	Yes (murine)	No	No	Low/No
NHP-FRG High Engraftment	No (NHP hepatocytes)	Yes (murine)	No	No	Low/No
NHP Cynomolgus Macaque	No	Yes (NHP)	Yes/Possible	Yes/Possible	Yes

	AAV Transduction Summary				
**Model**	**Top Ranked Variants Entry**	**Top Ranked Variants Expression**	**Bottom Ranked Variants Entry**	**Bottom Ranked Variants Expression**	**Figure**
Liver Explant	SYD12, LK03	SYD12, LK03	AAV9, FT11	FT11, AAV9	3
Liver Explant + Neutralizing Antibodies	AAV5, AAV8-Swap03	AAV8-Swap03, AAV8-Swap06	LK03, SEQ3	LK03-REDH,FT01	4
HuH-7	LK03, SEQ3	LK03, SEQ3	FT01, FT11	FT01, AAV5	S5
Naïve FRG	AAV5, AAV8	AAV8-Swap03, AAV8	LK03, SEQ3	FT11, FT01	S28
Humanized FRG Low	SYD12, SYD11	SYD12,SYD11	AAV8-Swap06, AAV5	SEQ3, AAV5	S28
Humanized FRG Mid	SYD12, SYD11	SYD12, SYD11	AAV8-Swap06, AAV5	SEQ3, AAV5	S28
Humanized FRG High	LK03-HSPG-,hu.Lvr06	SYD12, SYD11	AAV8-Swap06, AAV5	SEQ3, AAV5	S28
Humanized FRG High (2 months)	hu.Lvr06, LK03-REDH	hu.Lvr06, LK03-REDH	AAV8-Swap06, AAV5	AAV8-Swap06, AAV5	S29
NHP-FRG High Engraftment	hu.Lvr06, LK03-REDH	hu.Lvr06, LK03-REDH	FT11, AAV9	AAV9, AAV5	S27
NHP Cynomolgus Macaque	Swa05, SEQ3	Swap05, SEQ3	FT11, FT01	FT11, FT01	S26

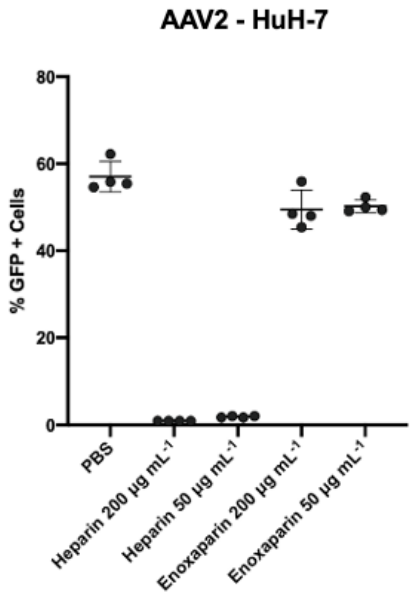

Supplementary Figure 1. Heparin and enoxaparin competition assay. Cells were transduced at 5,000 vector genomes/cell. When indicated, heparin sodium salt or enoxaparin were supplemented at 50 or 200 µg mL^{-1}. After 72 h, the cells were harvested and analyzed for Green Fluorescent Protein (GFP) expression, as indicated on the y-axis

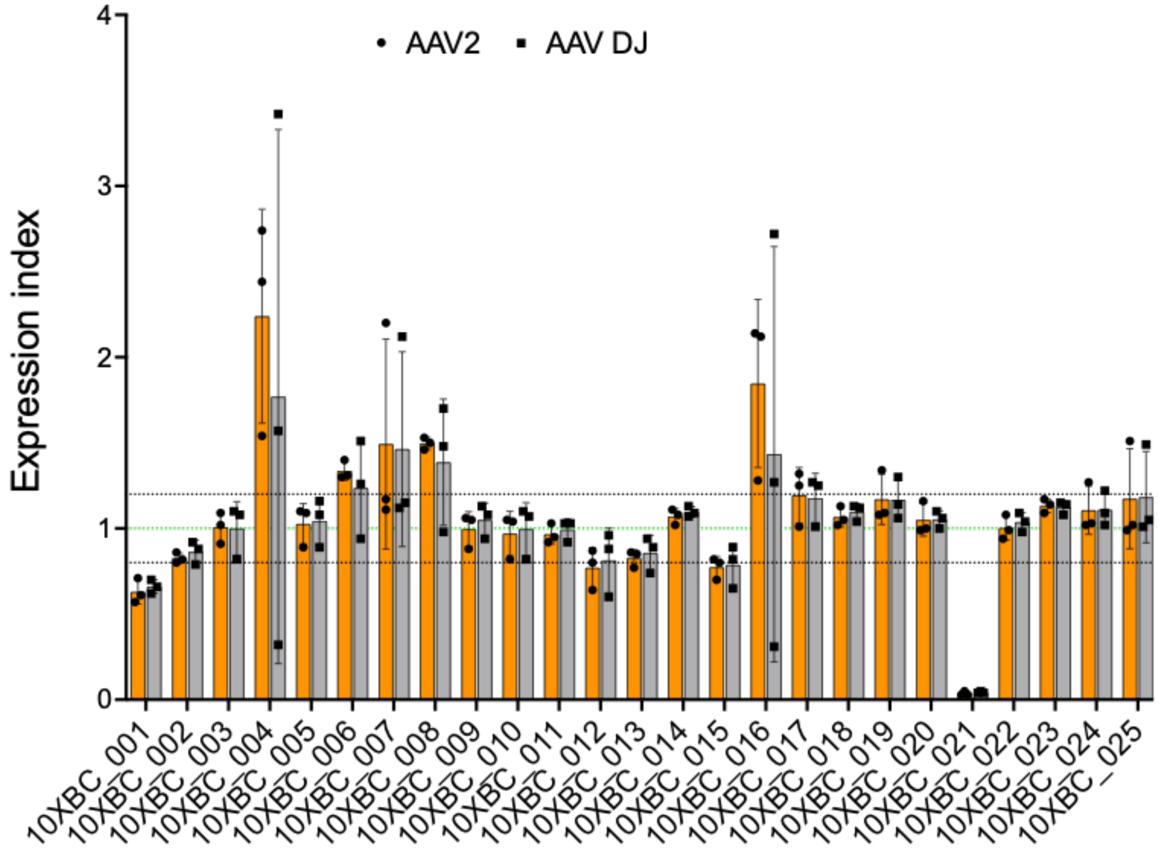

Supplementary Figure 2. RNA Stability test of the barcoded transgenes. The expression index refers to the quotient between Next-Generation Sequencing (NGS) reads mapped at the cDNA level and NGS reads mapped at the DNA level, for each independent barcode. To minimize a possible bias in transgene RNA stability introduced by the longer barcodes, the 25 individual barcoded constructs were packaged in AAV2 and AAV-DJ, and those constructs leading to either overexpression or under expression of the transgene when compared to the mean transduction values were discarded. Briefly, the 25 barcoded transgenes we were mixed at 1:1 molar ratio and co-transfected into HEK293T as described in the **Material and Methods** section for vector production. After AAV2 and AAV-DJ vector purification, 293T cells were transduced at three multiplicities of transduction (MOT) of 50, 500, and 5,000 vector genomes per cell. Three days after transduction, cells were harvested and the barcoded region was PCR-amplified from the vector preparation, DNA and RNA extracted from the cells. The results presented herein show the quotient of NGS reads from mRNA and gDNA. N=3 multiplicities of transduction (n=1 per MOT). The green highlighted barcodes were further validated (**Supplementary Fig. 3**).

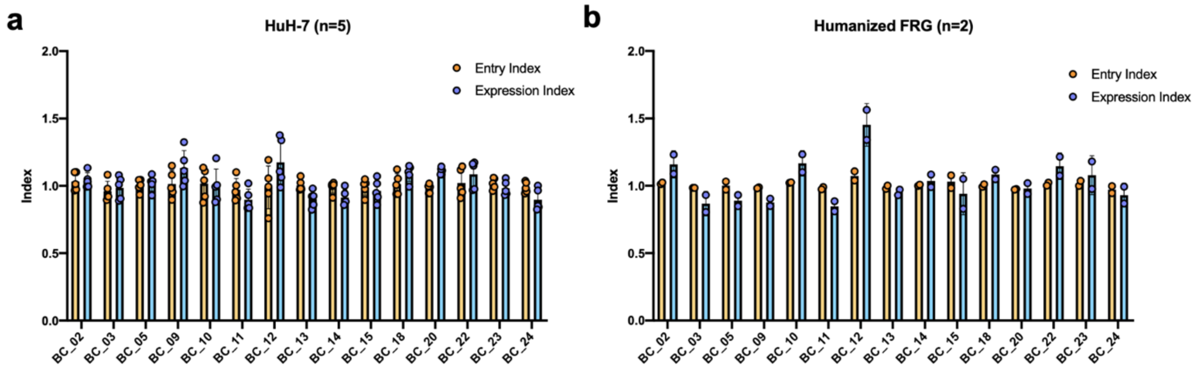

Supplementary Figure 3. RNA Stability test of the barcoded transgenes. To further validate the 15 chosen individual barcoded constructs, we co-packaged them in AAV-SYD12. **a,** Quotient of percentual NGS reads mapped to each barcoded transgene at the mRNA and at the gDNA level (Expression index). HuH-7 cells were transduced at MOT of 10,000 vector genomes per cell, and three days after transduction, cells were harvested and the barcoded region was PCR-amplified and sent for NGS analysis. **b**, Expression index of the same barcoded transgenes in human hepatocytes. The same vector preparation harboring the fifteen barcoded transgenes packaged into AAV-SYD12 was injected (5×10^{10} total vector genomes) into n=2 humanized FRG mice. Human hepatocytes were harvested one-week post-transduction and the barcoded region was PCR amplified from DNA and RNA extracted from the cells (**Material and Methods)** and sent for NGS analysis. Fourteen validated barcodes were then packaged individually into each capsid described in **Supplementary Table 1**.

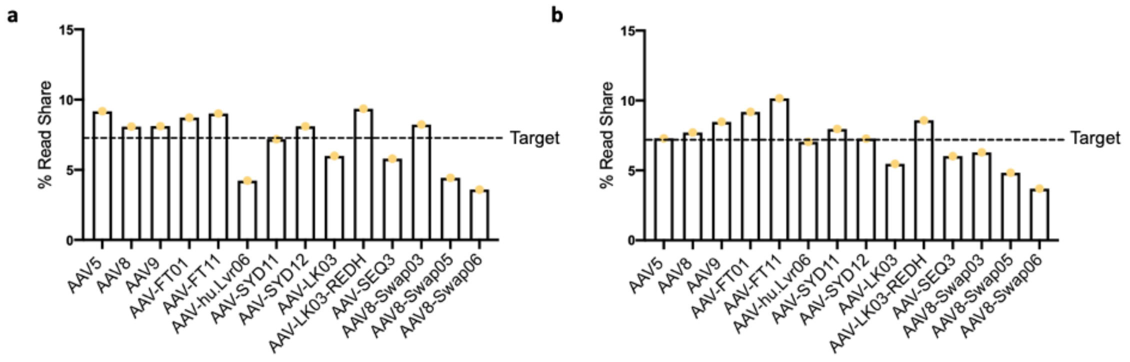

Supplementary Figure 4. Barcoded transgene pre-mix validation. Results show the percentage of NGS-reads mapped to each barcoded transgene packaged in the displayed AAV variants. The transgene was PCR amplified from the vector preparation. Given that fourteen capsids were present in the mix, the target equimolar distribution should contain 7.14% of each variant, indicated with a horizontal dashed line in the figure. **a,** Pre-mix validation injected to Donor 1. **b,** Pre-mix validation injected to Donor 2, mice and xenografted mice.

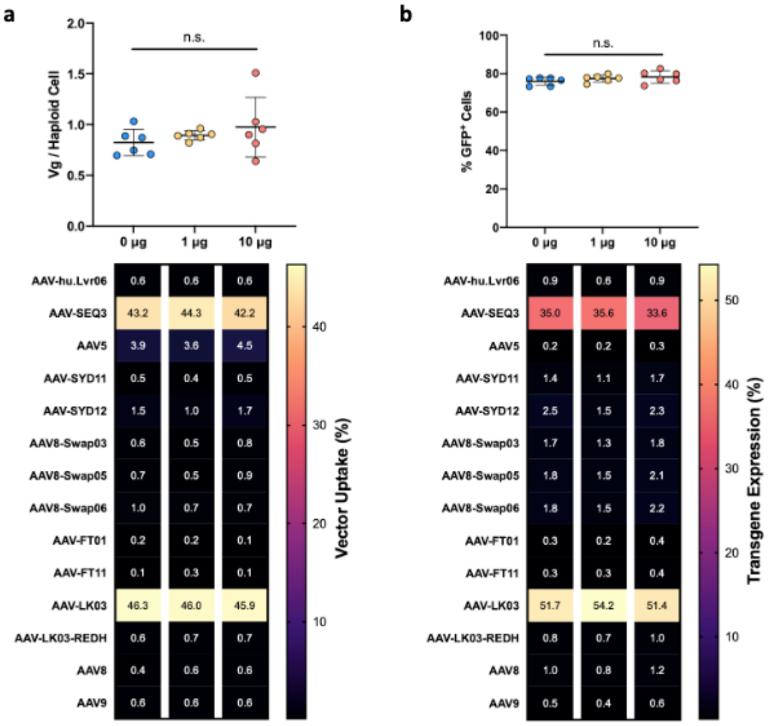

Supplementary Figure 5. Analysis of the *in vitro* influence of methylprednisolone on the transduction efficiency of AAV vectors. HuH-7 cells were transduced using the barcoded vector mix that contained 14 different AAV variants (10,000 vg/cell, n=6 per condition). Methylprednisolone was added to the culture medium in the amounts specified, either 1 or 10 µg (see **Methods** for details). **a**, the upper portion of the panel shows the vector copy number for the combined transgene mix, and the lower portion displays the NGS reads that correspond to each barcode-linked capsid at the DNA level, with normalization against the initial vector mix. **b**, transduction results; the upper portion of the panel shows proportion of cells expressing GFP at 72 hours post-transduction, and the bottom portion shows NGS reads aligned with each AAV vector based on barcode read at the cDNA level, normalized to the initial vector mix. The comparative analysis between different conditions revealed no significant disparities, suggesting that the presence of methylprednisolone at the specified concentrations does not markedly alter the relative transduction efficiency of the AAV vectors used in this work.

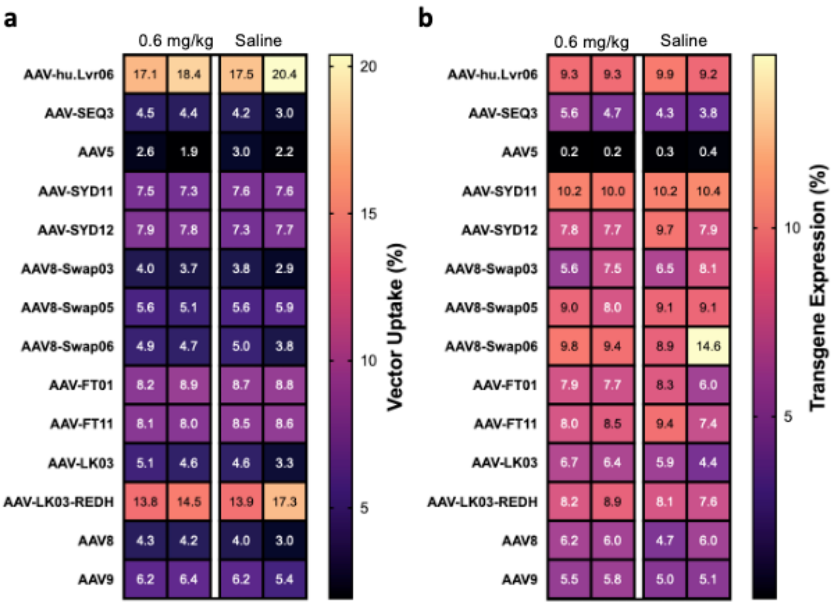

Supplementary Figure 6. *In vivo* effect of methylprednisolone on AAV transduction. a, to investigate how methylprednisolone impacts AAV-mediated transduction of human hepatocytes *in vivo*, we administered the vector mixture carrying 14 distinct barcoded capsids to four highly humanized FRG mice. The control group consisted of two mice receiving saline injections, while the test group included two mice treated with 0.6 mg/kg of methylprednisolone daily. Administration of methylprednisolone or saline commenced 24 hours before the introduction of the AAV vectors and continued for the subsequent four days. The mice were sacrificed on the fourth day following AAV administration, after which human hepatocytes were isolated following the procedure outlined in the **Methods** section. We amplified and sequenced the barcode region using NGS as detailed in **Methods** section. Results normalized to the vector mix for DNA (cell entry) and RNA (expression) are presented in panel **a** and **b**, respectively. Upon analysis, no major differences in transduction efficiency were noted between the methylprednisolone and control groups.

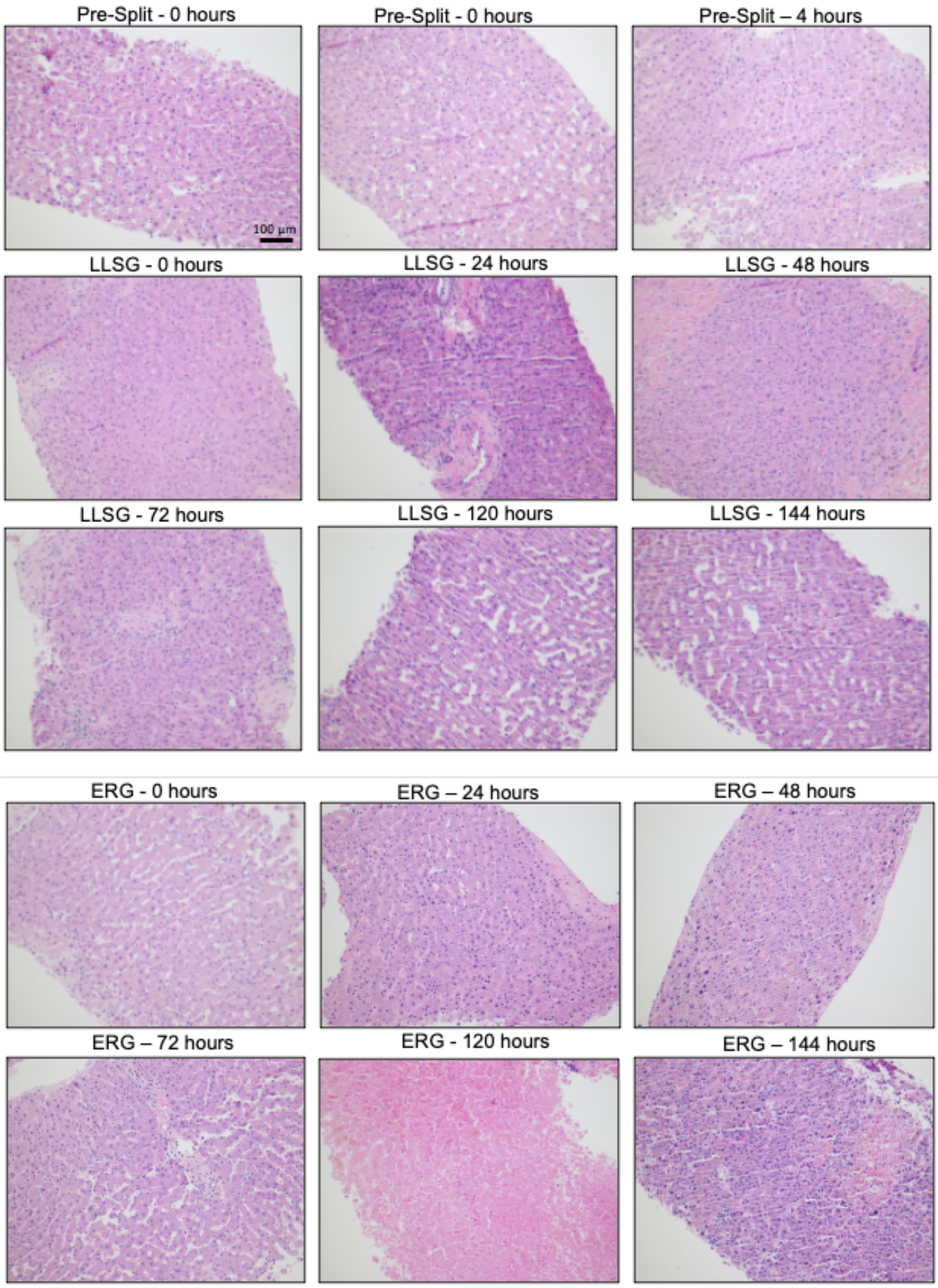

Supplementary Figure 7. Liver histology of representative core biopsies from Donor 1. Hematoxylin and eosin stain. LLSG – Left Lateral Sector Graft. ERG – Extended Right Graft. Scale: 100 µm. Images are representative from n=2 independent biopsies.

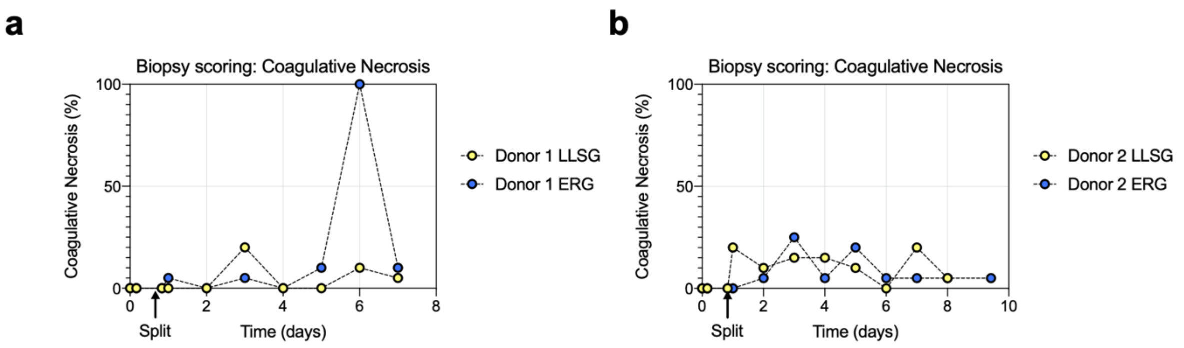

Supplementary Figure 8. Coagulative necrosis of livers (%). Core biopsies taken at the indicated time points were assessed by a specialist pathologist. **a,** Donor 1; **b,** Donor 2.

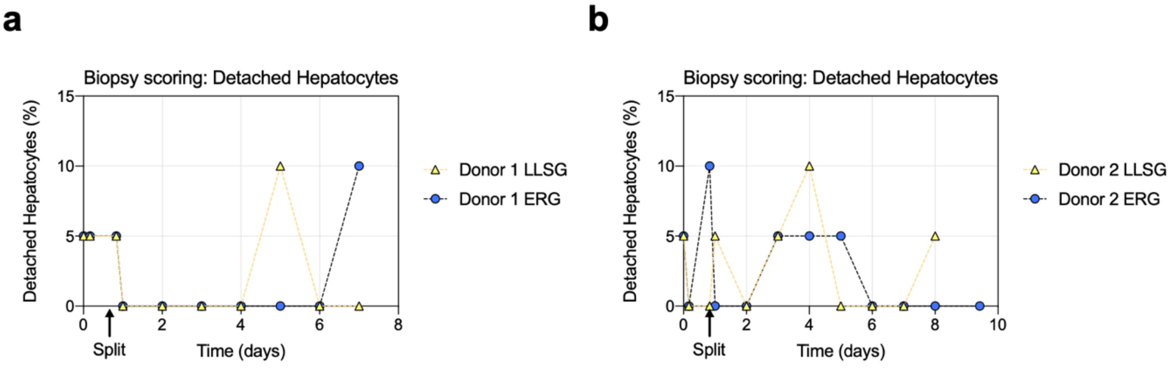

Supplementary Figure 9. Detached hepatocytes (%). Core biopsies taken at the indicated time points were assessed by a specialist pathologist. **a,** Donor 1; **b,** Donor 2.

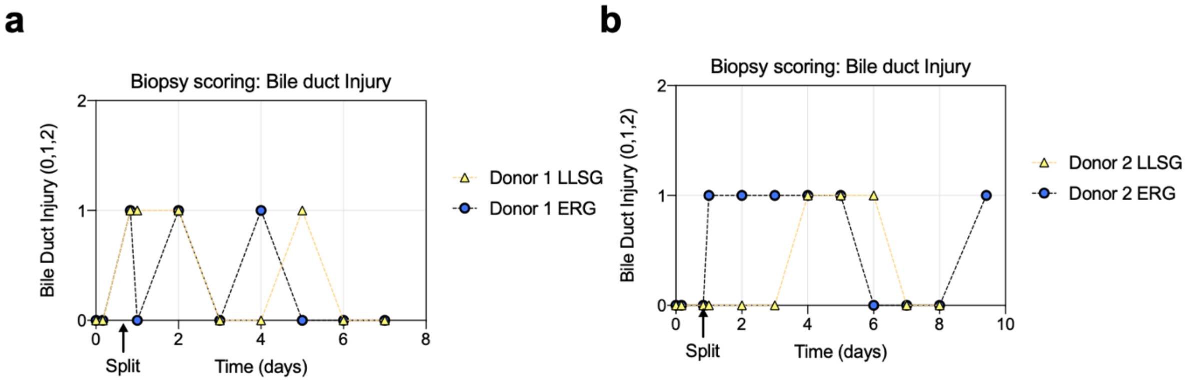

Supplementary Figure 10. Bile duct Injury. Core biopsies taken at the indicated time points were assessed by a specialist pathologist. **a,** Donor 1; **b,** Donor 2.

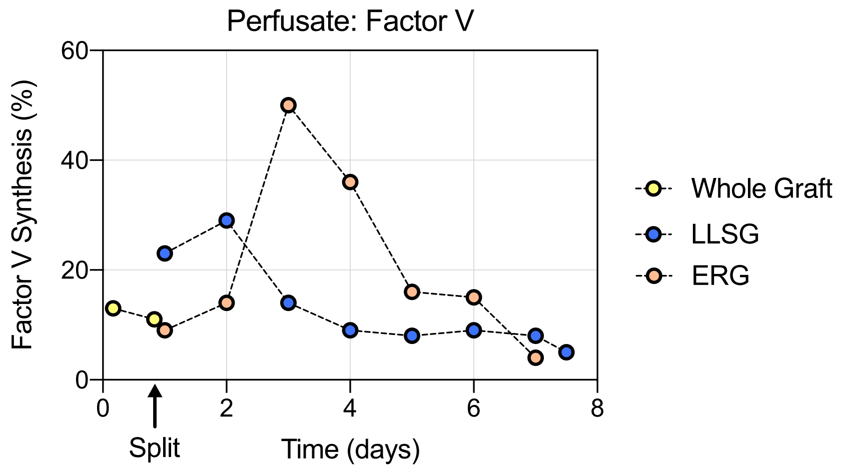

Supplementary Figure 11. Factor V synthesis. Course of Factor V synthesis for Donor 1, expressed as a percentage of the normal value, as studied *a posteriori* in samples taken from the perfusate throughout the perfusion. LLSG – Left Lateral Sector Graft. EG – Extended Right Graft.

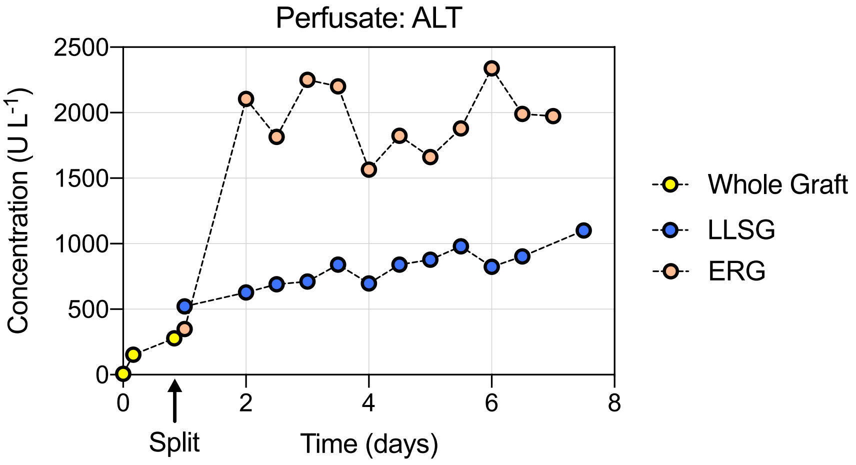

Supplementary Figure 12. Alanine aminotransferase (ALT) levels. Course of Alanine aminotransferase concentration in the perfusate for Donor 1, as studied *a posteriori* in samples taken from the perfusate throughout the perfusion. LLSG – Left Lateral Sector Graft. ERG – Extended Right Graft.

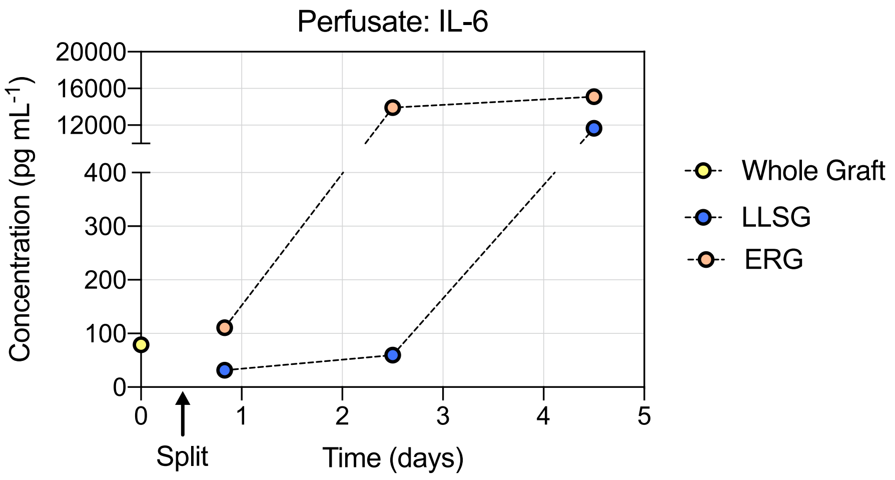

Supplementary Figure 13. Interleukin 6 (IL-6) levels. Course of IL-6 concentration in the perfusate for Donor 1, as studied *a posteriori* in samples taken from the perfusate throughout the perfusion. LLSG – Left Lateral Sector Graft. ERG – Extended Right Graft.

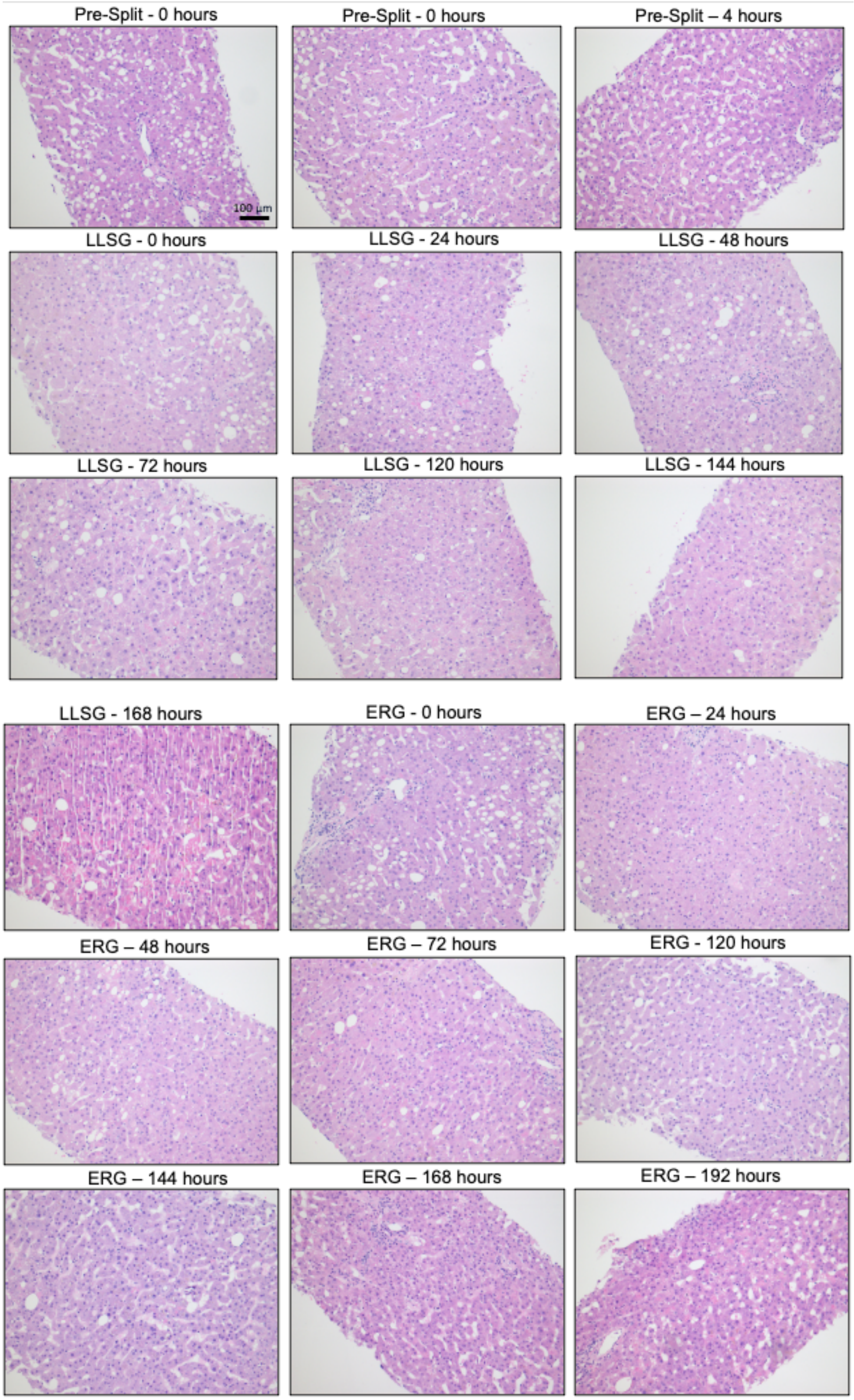

Supplementary Figure 14. Liver histology of representative core biopsies from Donor 2. Hematoxylin and eosin stain. LLSG – Left Lateral Sector Graft. ERG – Extended Right Graft. Scale: 100 µm. Images are representative from n=2 independent biopsies.

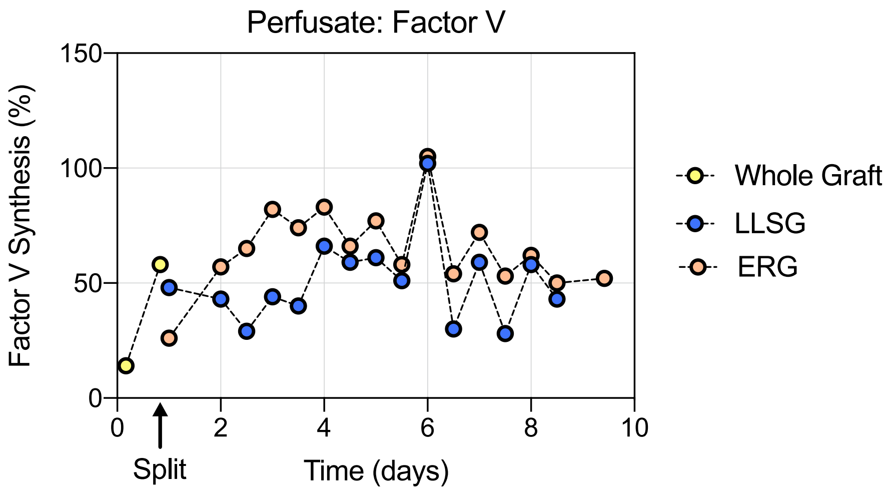

Supplementary Figure 15. Factor V synthesis. Course of Factor V synthesis for Donor 2, expressed as a percentage of the normal value, as studied *a posteriori* in samples taken from the perfusate throughout the perfusion. LLSG – Left Lateral Sector Graft. ERG – Extended Right Graft.

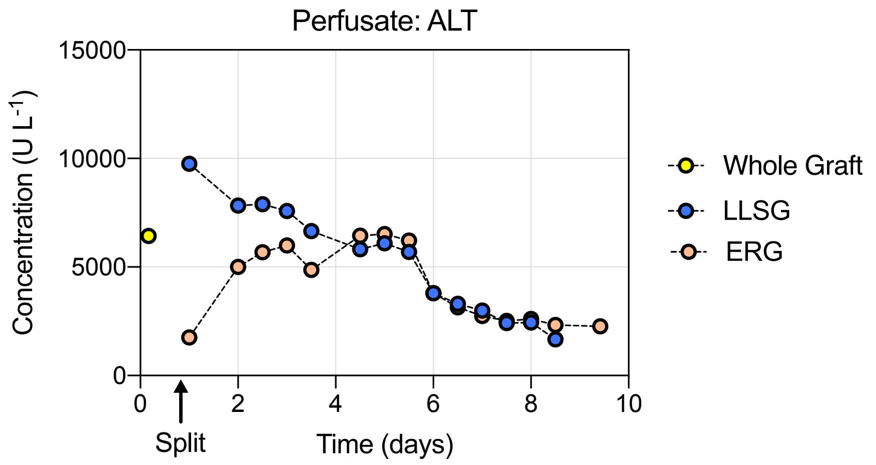

Supplementary Figure 16. Alanine aminotransferase (ALT) levels. Course of Alanine aminotransferase concentration in the perfusate for Donor 2, as studied *a posteriori* in samples taken from the perfusate throughout the perfusion. LLSG – Left Lateral Sector Graft. ERG – Extended Right Graft.

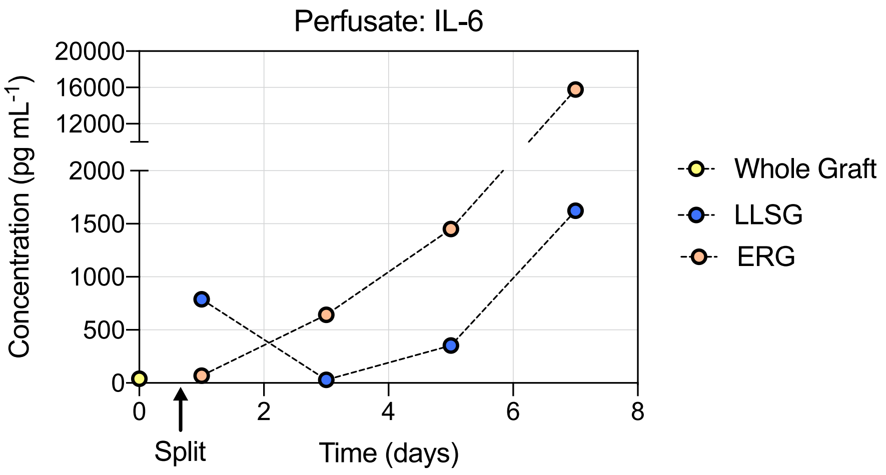

Supplementary Figure 17. Interleukin 6 (IL-6) levels. Course of IL-6 concentration in the perfusate for Donor 2, as studied *a posteriori* in samples taken from the perfusate throughout the perfusion. LLSG – Left Lateral Sector Graft. ERG – Extended Right Graft.

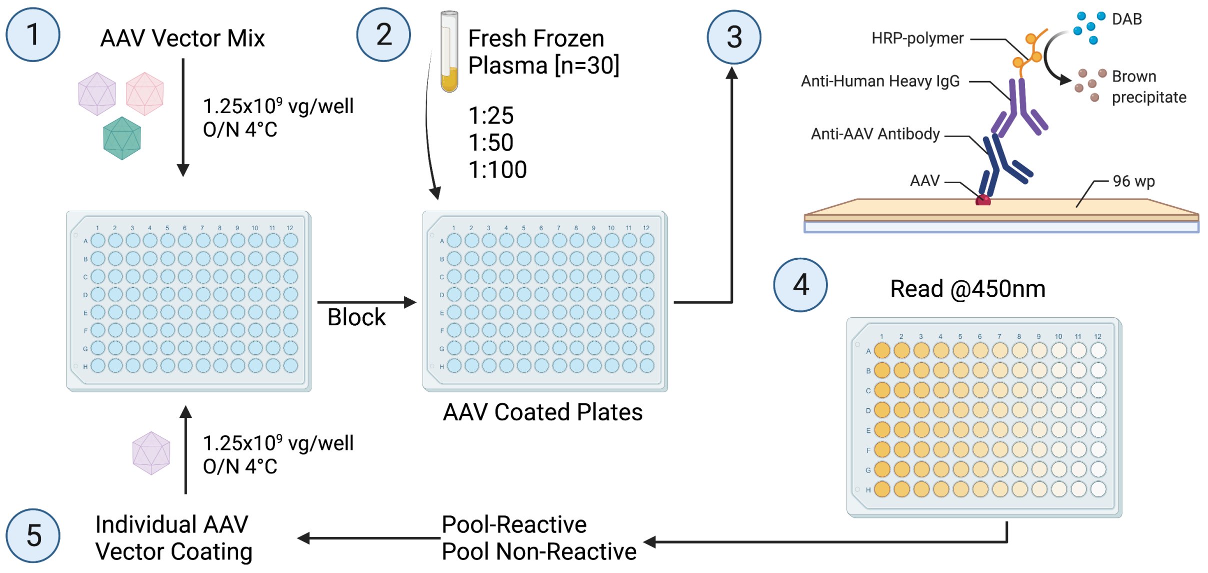

Supplementary Figure 18. ELISA method outline. Human sera were assayed for reactivity to all the fourteen capsids by ELISA, following a recently described method as outlined in the **Methods** section.

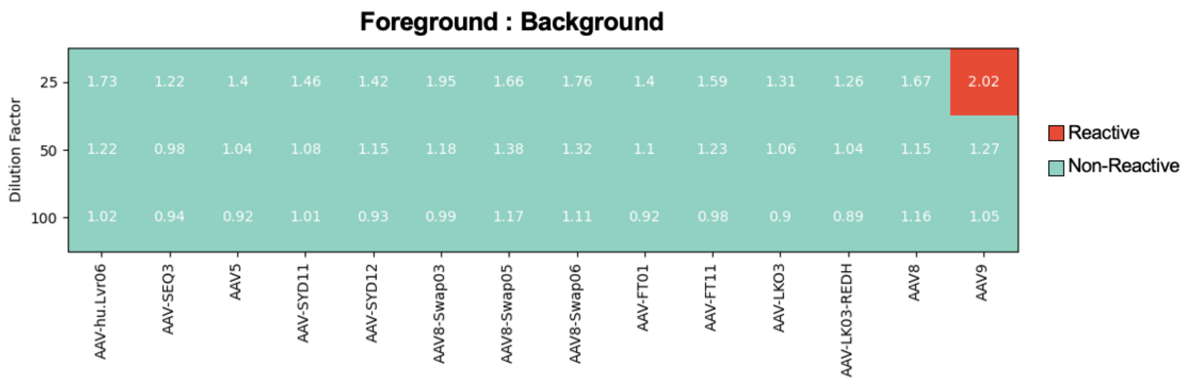

Supplementary Figure 19. Reactivity of the 'non-reactive' human plasma used in the perfusion of Donor 1. The mean value for each sample dilution was calculated for wells with (foreground) and without coated vector (background) and the sample was considered reactive if this ratio was >2.0. Only AAV9 was found to be slightly reactive at 1:25.

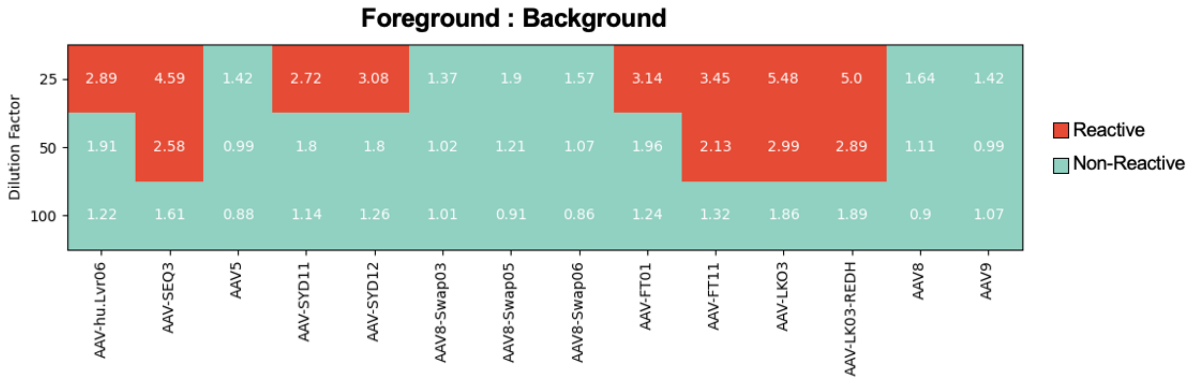

Supplementary Figure 20. Reactivity of the 'reactive' human plasma used in the perfusion of Donor 2. The mean value for each sample dilution was calculated for wells with (foreground) and without coated vector (background) and the sample was considered reactive if this ratio was >2.0.

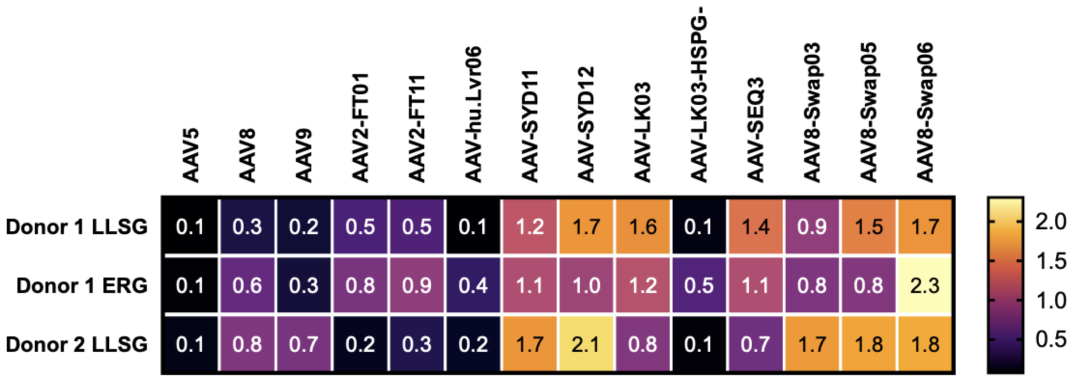

Supplementary Figure 21. Expression Index. Quotient of NGS reads from cDNA and viral gDNA from host cells. Values show the expression index at the last studied day for each liver (Day 5 and 8 post-transduction, respectively, for Donor 1 and 2).

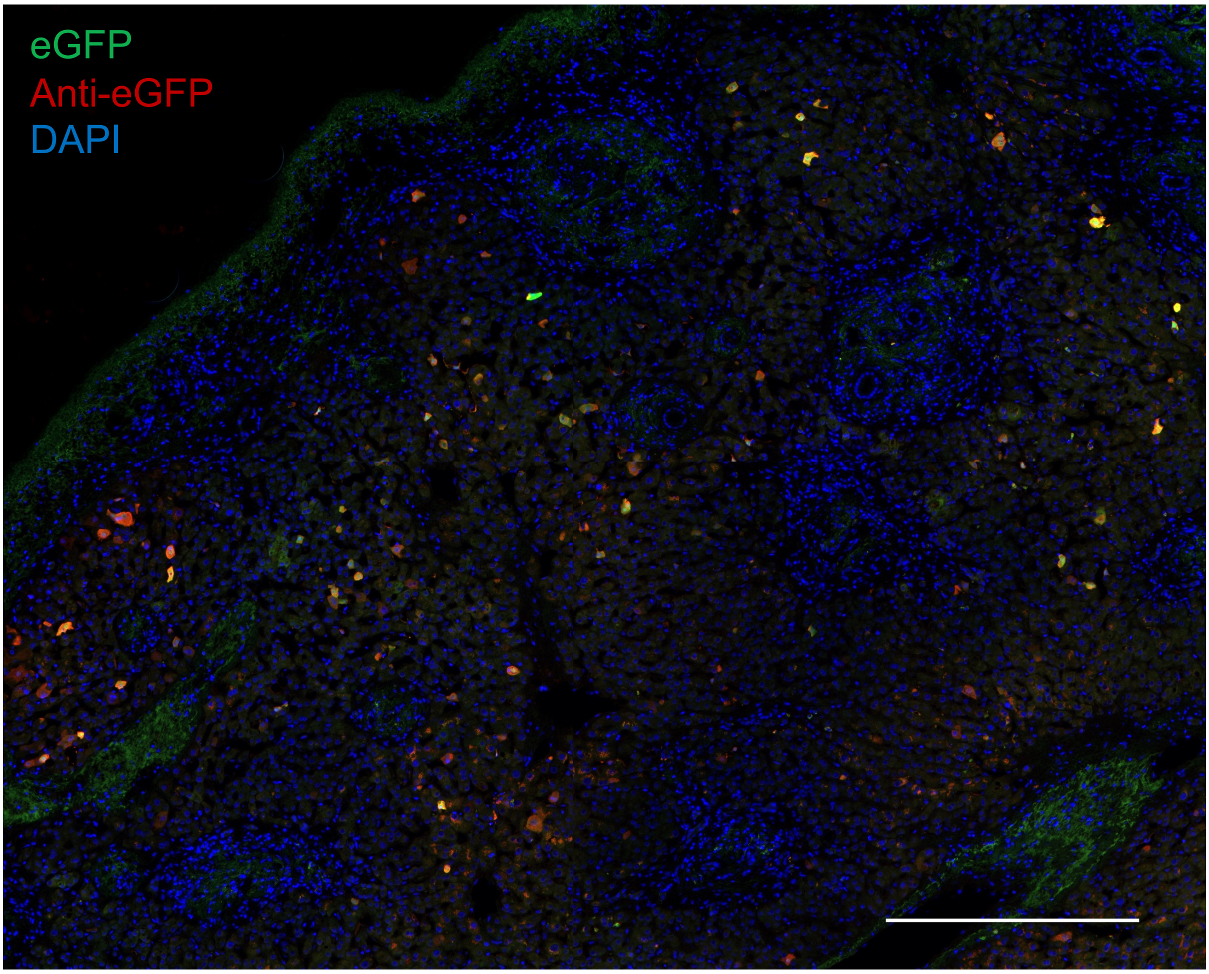

Supplementary Figure 22. Net eGFP signal. Representative immunofluorescence analysis of the net eGFP signal from collective AAV transduction in the left lobe of Donor 1 at day 4 post-transduction. The vector-encoded eGFP was also counterstained with an anti-eGFP antibody (red). Blue: DAPI (nuclei). Scale: 500 µm.

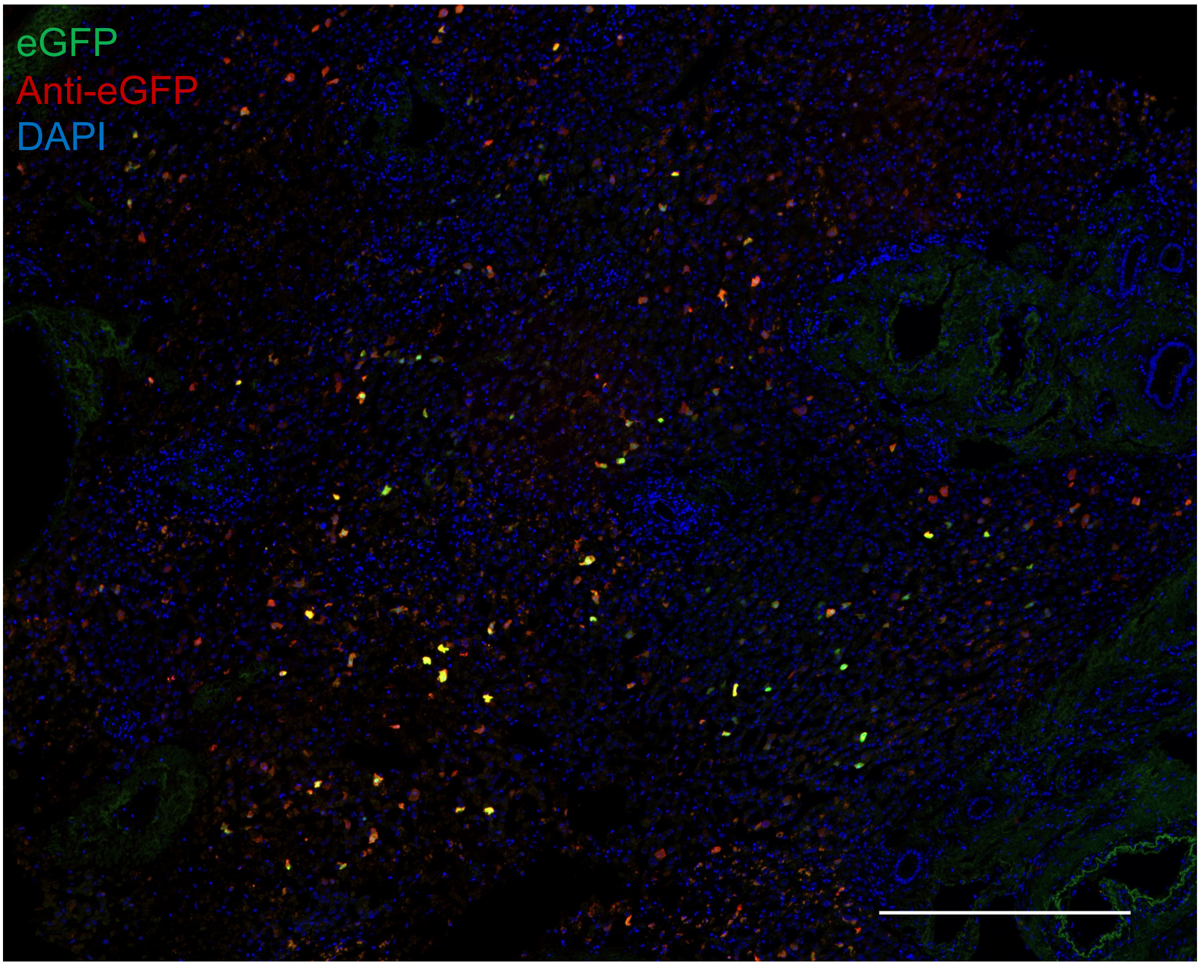

Supplementary Figure 23. Net eGFP signal. Representative immunofluorescence analysis of the net eGFP signal from collective AAV transduction in the right lobe of Donor 1 at day 4 post-transduction. The vector-encoded eGFP was also counterstained with an anti-eGFP antibody (red). Blue: DAPI (nuclei). Scale: 500 µm.

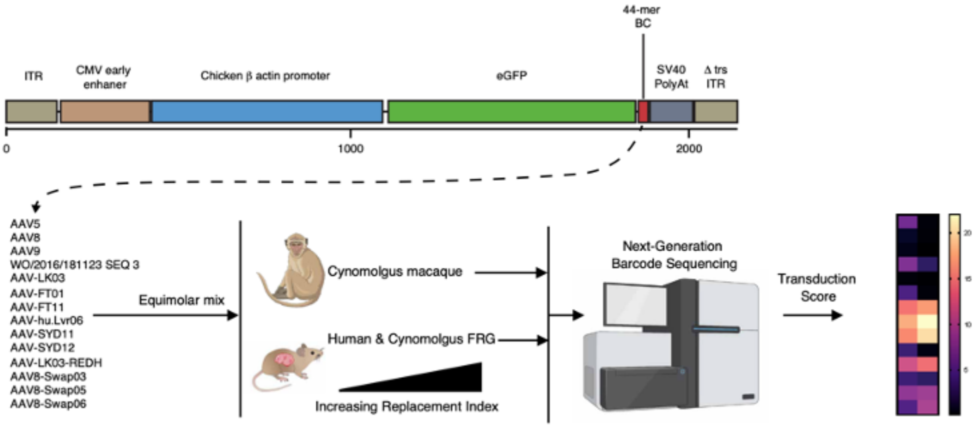

Supplementary Fig. 24. a, Functional evaluation of AAV vectors in murine and non-human primate liver models. To evaluate the results observed in the human liver explant in the context of other established preclinical models of human liver, we studied the relative performance of the same set of fourteen vectors (**Supplementary Table 1**) in the murine liver, the xenograft mouse models engrafted with human and non-human primate hepatocytes, as well as *in vivo* in a non-human primate (NHP). CMV - Cytomegalovirus; ITR – Inverted Terminal Repeat.

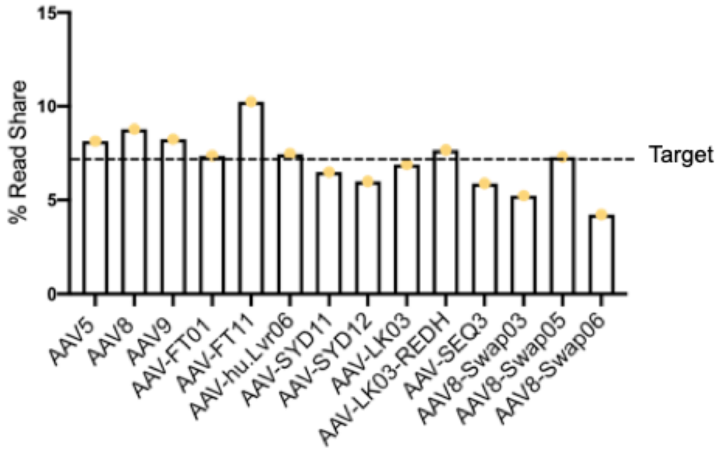

Supplementary Figure 25. Results show the percentage of NGS-reads mapped to each barcoded transgene packaged in the displayed AAV variants injected in the non-human primate described in **Supplementary Fig. 26**. The transgene was amplified with PCR from the vector preparation. Given that fourteen capsids were present in the mix, the target equimolar distribution would contain 7.14% of each variant, indicated with a horizontal dashed line.

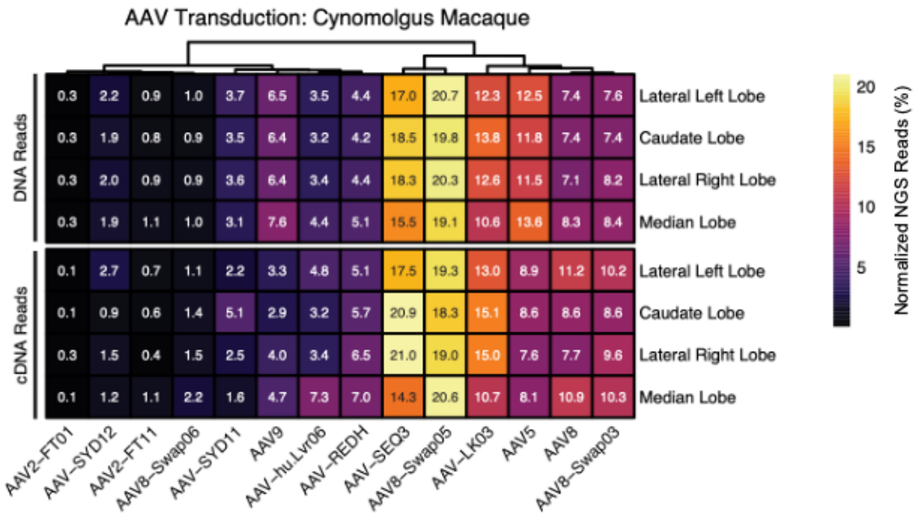

Supplementary Figure 26. ***In vivo*** **evaluation of the vector mix in the liver of a cynomolgus macaque NHP model.** Percentage of NGS reads mapped to each barcoded AAV capsid variant. On the top panel, the transgene DNA, indicating vector uptake, was extracted from tissue taken from the four indicated lobes one-month post-injection. The bottom panel shows a similar analysis performed on transgenes recovered from RNA, which indicate functional transduction. Percentages are normalized to the pre-injection mix. To minimize the influence of neutralizing antibodies, we removed the majority of antibodies using a previously validated immunoadsorption method.[2] After antibody depletion, the end titer, calculated for AAV9, was 1:30 (**Methods**). In contrast to the ELISA-based method that we used to assay human sera, a luciferase transduction assay was used in this case.[3] We then intravenously infused a total dose of 3.5×10^{12} vector genomes (1.4×10^{12} vg kg^{-1}) of a new barcoded AAV mix (**Supplementary Fig. 25**). One-month post injection, we analyzed the relative vector performance in the four liver lobes, both at the vector uptake (DNA) and transgene expression (mRNA/cDNA) levels using NGS of the unique barcoded region of the expression cassette. At this time point, the relative vector uptake was highly correlated with transgene expression ($r = 0.96$, $p = 4.98\times10^{-8}$, Spearman's correlation coefficient). As seen previously in the human liver explant, the variants that did not follow this pattern were AAV5, which showed efficient uptake but low transgene expression (12.37% vs 8.31% average read share, respectively, for AAV5), and to a lower extent AAV9. The AAV8 variant AAV8-Swap05 (containing AAV2's VR-I and AAV7's VRs VI-VIII) performed more efficiently among all vectors tested, both at cell entry and transgene expression, followed closely by the AAV3b related AAV-SEQ3 and AAV-LK03. In marked contrast to the data obtained in human liver explants (**Fig. 3**), the AAV-SYDs failed to efficiently transduce liver of this NHP.

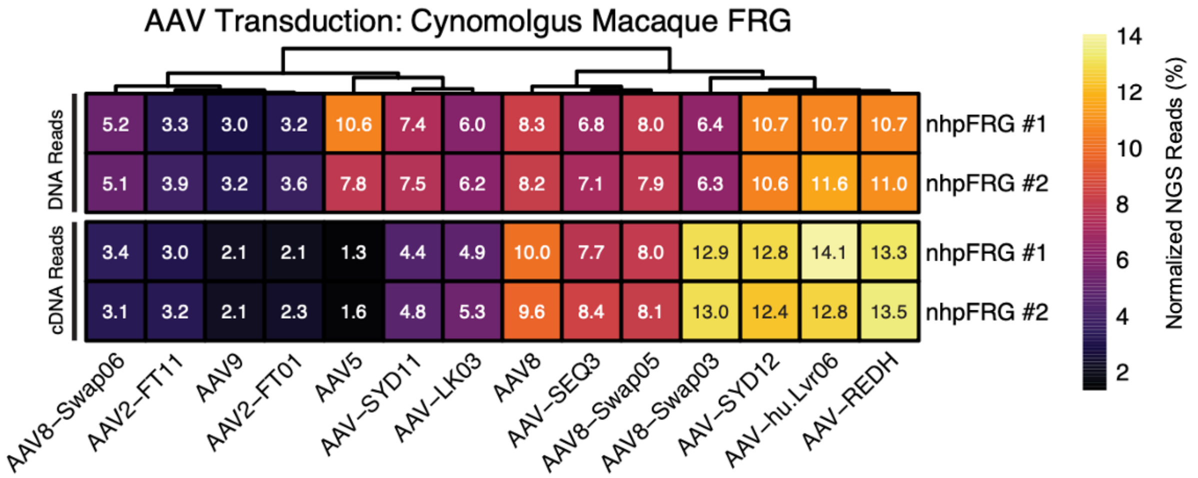

Supplementary Figure 27. ***In vivo*** **evaluation of the vector mix in FRG mice engrafted with cynomolgus macaque hepatocytes.** Percentage of NGS reads mapped to each barcoded AAV capsid variant. We evaluated the vector mix in a recently developed FRG xenograft murine model engrafted with primary hepatocytes from cynomolgus monkeys.[3] To do so, we injected highly engrafted animals (n=2) with albumin levels 10 mg mL^{-1} blood (equivalent to estimated >80% repopulation level) with 1×10^{11} total vg of the AAV mix (approximately 7.5×10^{9} vg per capsid) and harvested the chimeric livers two-weeks post-infusion. The results from this model resembled those from the NHP only for AAV5 and AAV9, where we observed the same post-entry defect for both serotypes. However, the top-performing variants in the cynomolgus model (AAV8-Swap05, AAV-SEQ3, AAV-LK03, **Supplementary Fig. 26**) ranked as medium performers in the xenograft mice engrafted with cynomolgus hepatocytes. Meanwhile, the top-ranking variants in the NHP-FRG xenograft model (AAV-LK03-REDH, AAV-hu.Lvr06 and AAV-SYD12) performed relatively poorly in the NHP model *in vivo* (**Supplementary Fig. 26**). These findings highlight important differences between the cynomolgus monkey NHP model and the xenograft murine model engrafted with primary hepatocytes from cynomolgus monkeys.

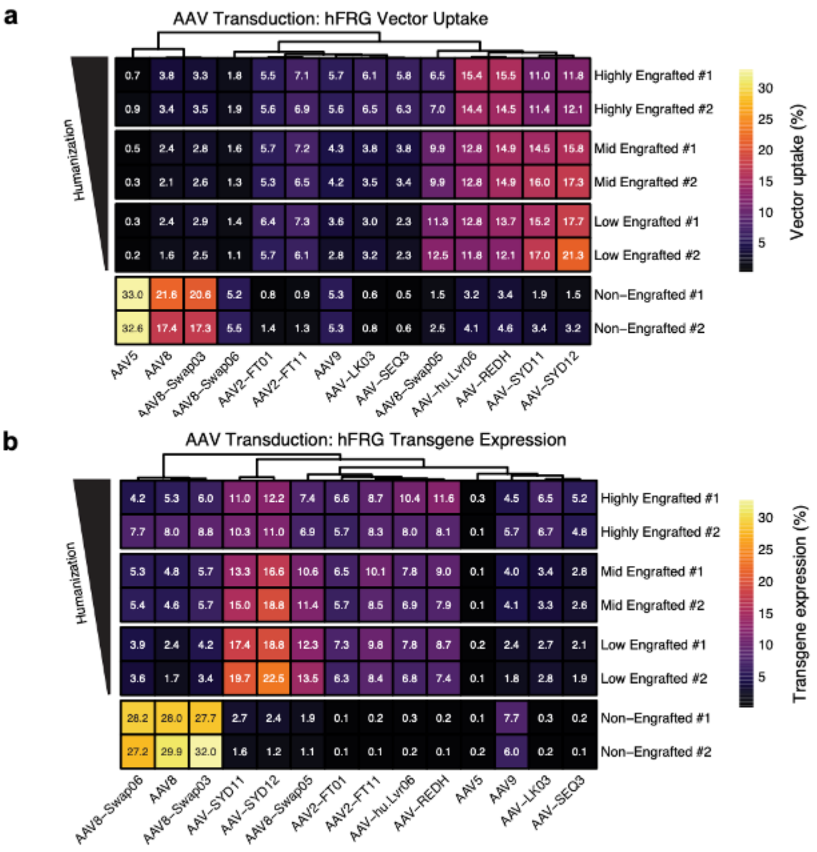

Supplementary Figure 28. ***In vivo*** **evaluation of the vector mix in naïve FRG mice and FRG mice engrafted with human hepatocytes. a,** Analyses at the DNA level (vector uptake) of sorted human hepatocytes performed in FRG presenting different levels of humanization as indicated by the black scale. **b,** Similar analyses at the cDNA level (transgene expression). For naïve FRG mice, the same analyses were carried out on DNA and RNA/cDNA extracted from the murine liver lysate. We investigated the same set of variants in humanized FRG models with various replacement indices (low ~10%, medium ~40% and high >80%, n=2 per repopulation level), as well as naïve (non-engrafted) FRGs. To facilitate direct comparison, we maintained the vector dose at a constant 7.5×10^9 vg per capsid (~1×10^{11} total vg) and harvested the mice one-week post vector injection. Relative AAV transduction in human hepatocytes displayed marked differences in comparison to the murine liver (non-engrafted animals). We found that AAV5, AAV8, and AAV-Swap03 efficiently transduced the murine liver, however, these same variants ranked poorly in human hepatocyte entry, irrespective of the level of engraftment. Our detailed analysis further revealed intriguing trends regarding vector performance in this preclinical human liver model. As the repopulation level increased (human-to-murine hepatocytes ratio), the relative function of AAV-SYDs and AAV8-Swap05 decreased. Conversely, the relative function of AAV-hu.Lvr06, AAV-LK03-REDH and the

HSPG-binders (AAV-LK03 and AAV-SEQ3) improved with an increase in the repopulation level. Overall, the AAV-SYDs, AAV-hu.Lvr06 and AAV-LK03-REDH performed the best in the humanized animals at vector uptake (**Panel a**). Regarding the transgene expression / functional transduction (RNA/cDNA), the results were even more pronounced in the murine liver, where only AAV8, AAV8-Swap03, and AAV8-Swap06 were able to achieve significant transgene expression. Consistent with the data obtained from other models, AAV5 showed poor transgene expression, even though it was highly efficient at entering murine hepatocytes Of note, in human hepatocytes, we also noted a relatively lower cDNA read share for AAV-LK03-REDH and AAV-hu.Lvr06 when contrasted to their performance at vector uptake, setting them apart, at least functionally, from the AAV-SYDs (**Panel b**).

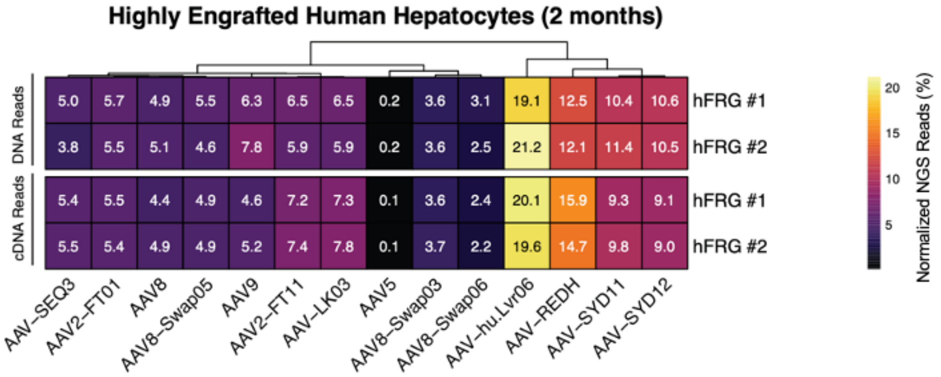

Supplementary Figure 29. Transduction of highly humanized FRG mice as analyzed two months after injection. Percentage of NGS reads mapped to each barcoded AAV capsid variant. On the top panel, the transgene DNA, indicating vector uptake, was extracted from sorted human hepatocytes, two months post-injection. The bottom panel shows a similar analysis performed on transgenes recovered from RNA, which indicate functional transduction. Percentages are normalized to the pre-injection mix. The correlation between cDNA and DNA was considerably higher at the two months' time point ($r = 0.94$, $p=1.8\times10^{-8}$) than at one-week post injection ($r = 0.79$, $p=8.0\times10^{-4}$) (**Supplementary Fig. 28**). This suggests that the expression kinetics, the aggregate result of the vector's entire intracellular journey, is an important and differing factor when evaluating relative vector function. Importantly, the correlation of DNA read share between both time points (one week vs two months) was also high ($r = 0.92$, $p=2.7\times10^{-8}$), further underlining the significance of considering the influence of vector trafficking kinetics on overall vector function.

Supplementary Note

In addition to performing functional vector comparison in the whole human liver explant and other established preclinical models, such as xenograft murine model and non-human primate, the data generated allowed us also to compare data from human liver explants to historical data garnered from other preclinical models. The comparison of AAV-LK03 and AAV8 might be the most relevant, given that clinical data for these vectors are publicly available in addition to data from multiple preclinical studies.[4,5] In the human explant under non-neutralizing conditions, AAV-LK03 surpassed AAV8 in both vector uptake and functional transduction (**Fig. 3d-e**). This aligns with cumulative clinical data for both capsids[4,5], however, it contradicts the hierarchical classification reported by Wang and colleagues in non-human primate studies[6], where AAV8 outperformed AAV-LK03. Interestingly, Li and colleagues reported that rAAV3b, which only differs by 8 amino acids from AAV-LK03, substantially outperformed rAAV8 in transducing NHP livers.[7] Our data are in line with those reported by Li *et al.* Specifically, when co-injected into a Cynomolgus monkey, both AAV-LK03 and the AAV3-like AAV-SEQ3 outperformed AAV8 at the level of vector uptake and transgene expression (**Supplementary Fig. 26**).

Interestingly, AAV-SYD12, which was the best performing variant at the level of transduction of the human liver explant in the absence of NAbs (**Fig. 3**), showed suboptimal performance in our NHP study (**Supplementary Fig. 26**), yet excelled targeting NHP primary hepatocytes within the FRG model (**Supplementary Fig. 27**). These findings suggest that AAV-SYD12's *in vivo* efficacy in NHP may be affected by organ competition, which is absent in the murine non-liver organs. Alternatively, or additionally, the performance could be influenced by the blockage of transduction in primates due to existing neutralizing antibodies (1:30 NAb titer to AAV9). To draw concrete conclusions, further investigations are warranted. Lastly, it should be noted that the efficacy of AAV-SYD12 in the FRG model engrafted with NHP hepatocytes may be artificially overestimated, which is another potential explanation for the observed performance differences across the various models.

It is our opinion that the studies performed in the xenograft FRG mice model highlight the impact that the level of human hepatocyte engraftment can have on the outcome of experiments. Data obtained with AAV-LK03 provide for a good exemplar to illustrate this point. AAV-LK03's performance varied depending on the degree of humanization, due to its high affinity for heparan sulfate proteoglycan (HSPG), a property contributing to a distinct

periportal transduction profile.[8] In low engrafted FRG mice, AAV-LK03 underperformed as fewer human hepatocyte clusters resided within the periportal zone. On the other hand, the relative performance of this capsid increased in highly engrafted mice where more human cells were randomly present within this zone. Using the human liver explant model, where all hepatocytes around the portal vein (AAVs entry route into the liver) are human, we found AAV-LK03's performance closely matched that observed in highly engrafted mice. Therefore, when evaluating AAV vectors' ability to transduce primary human hepatocytes, the use of highly engrafted animals could potentially ensure more accurate results.

It is important to state that the authors are not advocating replacement of the more tractable humanized FRG model with the whole human liver model. The FRG model has proven to be a robust preclinical tool, demonstrated by the successful development of vectors such as the AAV-SYDs and AAV-LK03 through directed evolution. However, we noted high discrepancies in transduction between the hFRG and human liver explant models for AAV-hu.Lvr06[9] and AAV-LK03-REDH.[8] Despite performing well in the humanized mouse model, these variants did not achieve the same level of performance in the liver explant model. This is specially intriguing for AAV-hu.Lvr06, a variant that was isolated directly from a human liver.[9] It is possible that the low HSPG attachment of AAV-hu.Lvr06 and AAV-LK03-REDH could free up more vectors for human cell uptake in the hFRG model, thereby artificially enhancing their bioavailability and overall relative performance. In the liver explant model, our data suggest these variants stay longer in the perfusate and performed better in the left graft of donor 1 where AAV was recirculated, providing more time for vector-cell interaction. In support of this, AAV-hu.Lvr06 outperformed all other variants in highly engrafted animals at two months post-injection, implying slower cell attachment/entry kinetics. Here it is important to note that our studies on vector performance in human livers explants were restricted to a roughly one-week timeframe. Given the observed dynamic nature of transgene expression observed in the FRG model, it is plausible that the relative functional transduction of the studied vectors, particularly at the RNA level, could exhibit variations with prolonged time frames. Therefore, while our data provides valuable insights into early vector performance and tissue tropism, future studies that examine these processes over more extended timeframes could further enrich our understanding of AAV vector behaviour and refine their use in gene therapy applications.

Supplementary References

1 Pillay, S. *et al.* Adeno-associated Virus (AAV) Serotypes Have Distinctive Interactions with Domains of the Cellular AAV Receptor. *J Virol* **91** (2017). https://doi.org:10.1128/JVI.00391-17

2 Salas, D. *et al.* Immunoadsorption enables successful rAAV5-mediated repeated hepatic gene delivery in nonhuman primates. *Blood Adv* **3**, 2632-2641 (2019). https://doi.org:10.1182/bloodadvances.2019000380

3 Westhaus, A. *et al.* Assessment of Pre-Clinical Liver Models Based on Their Ability to Predict the Liver-Tropism of Adeno-Associated Virus Vectors. *Hum Gene Ther* **34**, 273-288 (2023). https://doi.org:10.1089/hum.2022.188

4 Perrin, G. Q., Herzog, R. W. & Markusic, D. M. Update on clinical gene therapy for hemophilia. *Blood* **133**, 407-414 (2019). https://doi.org:10.1182/blood-2018-07-820720

5 George, L. A. *et al.* Multiyear Factor VIII Expression after AAV Gene Transfer for Hemophilia A. *N Engl J Med* **385**, 1961-1973 (2021). https://doi.org:10.1056/NEJMoa2104205

6 Wang, L. *et al.* Comparative Study of Liver Gene Transfer With AAV Vectors Based on Natural and Engineered AAV Capsids. *Mol Ther* **23**, 1877-1887 (2015). https://doi.org:10.1038/mt.2015.179

7 Li, S. *et al.* Efficient and Targeted Transduction of Nonhuman Primate Liver With Systemically Delivered Optimized AAV3B Vectors. *Mol Ther* **23**, 1867-1876 (2015). https://doi.org:10.1038/mt.2015.174

8 Cabanes-Creus, M. *et al.* Characterization of the humanized FRG mouse model and development of an AAV-LK03 variant with improved liver lobular biodistribution. *Mol Ther Methods Clin Dev* **28**, 220-237 (2023). https://doi.org:10.1016/j.omtm.2022.12.014

9 Cabanes-Creus, M. *et al.* Restoring the natural tropism of AAV2 vectors for human liver. *Sci Transl Med* **12** (2020). https://doi.org:10.1126/scitranslmed.aba3312
