# Supplementary material for: Harnessing whole human liver ex situ normothermic perfusion for preclinical AAV vector evaluation
Source: Nat Commun. 2024 Mar 14;15:1876. doi: 10.1038/s41467-024-46194-y (PMC10940703; doi:10.1038/s41467-024-46194-y)
Supplement: Supplementary file 2 — Reporting Summary [file 41467_2024_46194_MOESM2_ESM.pdf]

nature portfolio

Corresponding author(s): Leszek Lisowski

Last updated by author(s): Feb 14, 2024

Reporting Summary

Nature Portfolio wishes to improve the reproducibility of the work that we publish. This form provides structure for consistency and transparency in reporting. For further information on Nature Portfolio policies, see our Editorial Policies and the Editorial Policy Checklist.

Statistics

For all statistical analyses, confirm that the following items are present in the figure legend, table legend, main text, or Methods section.

n/a	Confirmed	
☐	☒	The exact sample size (*n*) for each experimental group/condition, given as a discrete number and unit of measurement
☐	☒	A statement on whether measurements were taken from distinct samples or whether the same sample was measured repeatedly
☐	☒	The statistical test(s) used AND whether they are one- or two-sided<br>*Only common tests should be described solely by name; describe more complex techniques in the Methods section.*
☒	☐	A description of all covariates tested
☒	☐	A description of any assumptions or corrections, such as tests of normality and adjustment for multiple comparisons
☐	☒	A full description of the statistical parameters including central tendency (e.g. means) or other basic estimates (e.g. regression coefficient) AND variation (e.g. standard deviation) or associated estimates of uncertainty (e.g. confidence intervals)
☐	☒	For null hypothesis testing, the test statistic (e.g. *F*, *t*, *r*) with confidence intervals, effect sizes, degrees of freedom and *P* value noted<br>*Give P values as exact values whenever suitable.*
☒	☐	For Bayesian analysis, information on the choice of priors and Markov chain Monte Carlo settings
☒	☐	For hierarchical and complex designs, identification of the appropriate level for tests and full reporting of outcomes
☐	☒	Estimates of effect sizes (e.g. Cohen's *d*, Pearson's *r*), indicating how they were calculated

Our web collection on statistics for biologists contains articles on many of the points above.

Software and code

Policy information about availability of computer code

Data collection	Relative AAV transduction was studied by amplifyingt he transgene region containing the unique 44-mer barcodes. NGS library preparations and sequencing using 2 × 150 paired-end configurations were performed by Azenta (Suzhou, China) using an Illumina NovaSeq instrument. NGS reads from the DNA and cDNA populations extracted from the targeted tissues were normalized to the reads from the pre-injection, vector mix.
Data analysis	A workflow was written in Snakemake (5.6) to process reads and count barcodes. Paired reads were merged using BBMerge and then filtered for reads of the expected length in a second pass through BBDuk, both from BBTools 38.68 (https://sourceforge.net/projects/bbmap/). The merged, filtered fastq files were passed to a Python (3.7) script that identified barcodes corresponding to AAV variants. All scripts used to count barcodes in paired-end Illumina reads from PCR amplicons are available on Code Ocean (https://doi.org/10.24433/CO.7176285.v1). Graphs, statistical analyses were generted using Prism software 8.4.2 (GraphPad),. Heat maps were generated with R studio with pheatmap package.

For manuscripts utilizing custom algorithms or software that are central to the research but not yet described in published literature, software must be made available to editors and reviewers. We strongly encourage code deposition in a community repository (e.g. GitHub). See the Nature Portfolio guidelines for submitting code & software for further information.

Data

Policy information about availability of data

All manuscripts must include a data availability statement. This statement should provide the following information, where applicable:

- Accession codes, unique identifiers, or web links for publicly available datasets
- A description of any restrictions on data availability
- For clinical datasets or third party data, please ensure that the statement adheres to our policy

All data generated or analysed during this study are included in this published article and the Supplementary information files. Raw sequencing data are available via accession code PRJNA1076589. Source data are provided with this paper. All the capsid sequences used in this article have been previously described in the literature (Supplementary Table 1). Any other relevant data are available from the corresponding author upon request.

Research involving human participants, their data, or biological material

Policy information about studies with human participants or human data. See also policy information about sex, gender (identity/presentation), and sexual orientation and race, ethnicity and racism.

Reporting on sex and gender	The sex, and age of the donors of the human livers are reported, as well as the sex of the donor of the human hepatocytes utilized to engraft FRG mice. All hFRG mice reported in this study were engrafted with human and non-human primate hepatocytes from the same donors (Lonza, #HUM181791; ThermoFisher, #CY409).
Reporting on race, ethnicity, or other socially relevant groupings	n/a
Population characteristics	Donor 1: Age: 78, Sex: F, Donor Type: Donation after brain death; Donor 2: Age 56, Sex: M, Donor Type: Donation after circulatory determination of death.
Recruitment	n/a
Ethics oversight	Human livers that were declined for transplantation but consented for research were obtained through the centralised donation organisation in Australia, DonateLife. These were accepted in accordance with a study protocol approved by the Sydney Local Health District Ethics Review Committee (X18-0523 & 2019/ETH08964).

Note that full information on the approval of the study protocol must also be provided in the manuscript.

Field-specific reporting

Please select the one below that is the best fit for your research. If you are not sure, read the appropriate sections before making your selection.

[x] Life sciences [ ] Behavioural & social sciences [ ] Ecological, evolutionary & environmental sciences

For a reference copy of the document with all sections, see nature.com/documents/nr-reporting-summary-flat.pdf

Life sciences study design

All studies must disclose on these points even when the disclosure is negative.

Sample size	Relative vector analyses were performed in two independent human livers. No statistical calculation was performed to choose sample size. A sample size calculation was not possible as there are no previous studies for comparison. We considered a sample size of 2 organs to be reasonable to produce a proof of concept of this model for AAV evaluation. Biological replicates (N=2) were performed in murine studies, and N=1 for the NHP study.
Data exclusions	No data were excluded from analyses.
Replication	Except for the non-human primate study, each experiment was performed at least two times with no issues with reproducibility. For the non-human primate study, four independent biopsies were taken from the same liver, in order to verify the reproducibility of the experimental findings.<br>The ELISA method described herein was performed in two technical replicates and all attempts at replication were successful.
Randomization	Not relevant. Single intervention group.
Blinding	Investigators were not blinded during monitoring and transduction evaluation of AAVs. Blinding not relevant, single intervention groups.

Reporting for specific materials, systems and methods

We require information from authors about some types of materials, experimental systems and methods used in many studies. Here, indicate whether each material, system or method listed is relevant to your study. If you are not sure if a list item applies to your research, read the appropriate section before selecting a response.

Materials & experimental systems

n/a	Involved in the study
☐	☒ Antibodies
☐	☒ Eukaryotic cell lines
☒	☐ Palaeontology and archaeology
☐	☒ Animals and other organisms
☒	☐ Clinical data
☒	☐ Dual use research of concern
☒	☐ Plants

Methods

n/a	Involved in the study
☒	☐ ChIP-seq
☒	☐ Flow cytometry
☒	☐ MRI-based neuroimaging

Antibodies

Antibodies used

Anti-eGFP (Invitrogen, #A-11122, 1:100), Anti-HNF4 (Abcam, #ab41898, 1:100), Anti-HAL (Sigma Prestige Antibodies, #HPA038547, 1:100), anti-Cytokeratin 7 (Abcam, #ab68459, 1:100), anti-albumin (Bethyl, #A80229A, 1:100), anti-KIAA0319L (AAVR, Abcam, #ab105385, 1:100), anti-CD68 (Invitrogen, #14068882, 1:100), phycoerythrin (PE)-conjugated anti-human-HLA-ABC (clone W6/32, Invitrogen 12-9983-42, 1:20), anti-mouse-H-2Kb (clone AF6-88.5, BD Pharmigen 553,568, 1:100), and allophycocyanin (APC)-conjugated streptavidin (eBioscience 17-4317-82, 1:500) antibodies were used.

Validation

Anti-eGFP (Invitrogen, #A-11122). This Antibody was verified by Relative expression to ensure that the antibody binds to the antigen stated. https://www.thermofisher.com/antibody/product/GFP-Antibody-Polyclonal/A-11122 Chen L, Li Y, Wu Y, Li S, Chang X, Wu H. Protocol for analysis of senescent neuronal stem cells in genetic-modified embryonic mice using in utero electroporation technique. STAR Protoc. 2022 Jun 13;3(3):101461. doi: 10.1016/j.xpro.2022.101461. PMID: 35719723; PMCID: PMC9201042.

Anti-HNF4alpha(Abcam, #ab41898). Validated for IF in HepG2 cells. Huang L, Yang Q, Chen H, Wang Z, Liu Q, Ai S. Tollip promotes hepatocellular carcinoma progression via PI3K/AKT pathway. Open Med (Wars). 2022 Apr 1;17(1):626-637. doi: 10.1515/med-2022-0453. Erratum in: Open Med (Wars). 2022 Jun 11;17(1):1065. PMID: 35434373; PMCID: PMC8976180.

Anti-HAL (Sigma Prestige Antibodies, #HPA038547). Validated in our previous work. Cabanes-Creus M, Navarro RG, Liao SHY, Scott S, Carlessi R, Roca-Pinilla R, Knight M, Baltazar G, Zhu E, Jones M, Denisenko E, Forrest ARR, Alexander IE, Tirnitz-Parker JEE, Lisowski L. Characterization of the humanized FRG mouse model and development of an AAV-LK03 variant with improved liver lobular biodistribution. Mol Ther Methods Clin Dev. 2023 Jan 2;28:220-237. doi: 10.1016/j.omtm.2022.12.014. PMID: 36700121; PMCID: PMC9860073.

Anti-Cytokeratin 7 (Abcam, #ab68459). Validated in our previous work. Cabanes-Creus M, Navarro RG, Liao SHY, Scott S, Carlessi R, Roca-Pinilla R, Knight M, Baltazar G, Zhu E, Jones M, Denisenko E, Forrest ARR, Alexander IE, Tirnitz-Parker JEE, Lisowski L. Characterization of the humanized FRG mouse model and development of an AAV-LK03 variant with improved liver lobular biodistribution. Mol Ther Methods Clin Dev. 2023 Jan 2;28:220-237. doi: 10.1016/j.omtm.2022.12.014. PMID: 36700121; PMCID: PMC9860073.

Anti-albumin (Bethyl, #A80229A). Validated in Mancio-Silva L, Fleming HE, Miller AB, Milstein S, Liebow A, Haslett P, Sepp-Lorenzino L, Bhatia SN. Improving Drug Discovery by Nucleic Acid Delivery in Engineered Human Microlivers. Cell Metab. 2019 Mar 5;29(3):727-735.e3. doi: 10.1016/j.cmet.2019.02.003. PMID: 30840913; PMCID: PMC6408324.

Anti-KIAA0319L (AAVR, Abcam, #ab105385). Validated in our previous work. Cabanes-Creus M, Westhaus A, Navarro RG, Baltazar G, Zhu E, Amaya AK, Liao SHY, Scott S, Sallard E, Dilworth KL, Rybicki A, Drouyer M, Hallwirth CV, Bennett A, Santilli G, Thrasher AJ, Agbandje-McKenna M, Alexander IE, Lisowski L. Attenuation of Heparan Sulfate Proteoglycan Binding Enhances In Vivo Transduction of Human Primary Hepatocytes with AAV2. Mol Ther Methods Clin Dev. 2020 May 13;17:1139-1154. doi: 10.1016/j.omtm.2020.05.004. PMID: 32490035; PMCID: PMC7260615.

Anti-CD68 (Invitrogen, #14068882). Golden GJ, Toledo AG, Marki A, Sorrentino JT, Morris C, Riley RJ, Spliid C, Chen Q, Cornax I, Lewis NE, Varki N, Le D, Malmström J, Karlsson C, Ley K, Nizet V, Esko JD. Endothelial Heparan Sulfate Mediates Hepatic Neutrophil Trafficking and Injury during Staphylococcus aureus Sepsis. mBio. 2021 Oct 26;12(5):e0118121. doi: 10.1128/mBio.01181-21. Epub 2021 Sep 21. PMID: 34544271; PMCID: PMC8546592.

phycoerythrin (PE)-conjugated anti-human-HLA-ABC (clone W6/32, Invitrogen 12-9983-42; 1:20). Validated in our previous work. Cabanes-Creus M, Navarro RG, Liao SHY, Scott S, Carlessi R, Roca-Pinilla R, Knight M, Baltazar G, Zhu E, Jones M, Denisenko E, Forrest ARR, Alexander IE, Tirnitz-Parker JEE, Lisowski L. Characterization of the humanized FRG mouse model and development of an AAV-LK03 variant with improved liver lobular biodistribution. Mol Ther Methods Clin Dev. 2023 Jan 2;28:220-237. doi: 10.1016/j.omtm.2022.12.014. PMID: 36700121; PMCID: PMC9860073.

anti-mouse-H-2Kb (clone AF6-88.5, BD Pharmigen 553,568; 1:100). Validated in our previous work. Cabanes-Creus M, Navarro RG, Liao SHY, Scott S, Carlessi R, Roca-Pinilla R, Knight M, Baltazar G, Zhu E, Jones M, Denisenko E, Forrest ARR, Alexander IE, Tirnitz-Parker JEE, Lisowski L. Characterization of the humanized FRG mouse model and development of an AAV-LK03 variant with improved liver lobular biodistribution. Mol Ther Methods Clin Dev. 2023 Jan 2;28:220-237. doi: 10.1016/j.omtm.2022.12.014. PMID: 36700121;

PMCID: PMC9860073.

allophycocyanin (APC)-conjugated streptavidin (eBioscience 17-4317-82; 1:500). Validated in our previous work. Cabanes-Creus M, Navarro RG, Liao SHY, Scott S, Carlessi R, Roca-Pinilla R, Knight M, Baltazar G, Zhu E, Jones M, Denisenko E, Forrest ARR, Alexander IE, Tirnitz-Parker JEE, Lisowski L. Characterization of the humanized FRG mouse model and development of an AAV-LK03 variant with improved liver lobular biodistribution. Mol Ther Methods Clin Dev. 2023 Jan 2;28:220-237. doi: 10.1016/j.omtm.2022.12.014. PMID: 36700121; PMCID: PMC9860073.

Eukaryotic cell lines

Policy information about cell lines and Sex and Gender in Research

Cell line source(s)	HuH-7 cells were kindly provided by Dr Jerome Laurence (The University of Sydney). HEK293T cells were obtained from ATCC (Cat#CRL-3216).
Authentication	HEK293T and Huh-7 cells were not authenticated.
Mycoplasma contamination	All cells were tested for mycoplasma and were mycoplasma-free.
Commonly misidentified lines (See ICLAC register)	No commonly misidentified cell lines were used in this study.

Animals and other research organisms

Policy information about studies involving animals; ARRIVE guidelines recommended for reporting animal research, and Sex and Gender in Research

Laboratory animals	Fah-/-Rag2−/−Il2rg-/- (FRG) mice were bred, housed (12 hours dark/light; ~50% humidity; 22-23°C), engrafted at 6 to 8 weeks of age, and monitored at the Bioresources Core of the Children's Medical Research Insitute. Levels of human and non-human primate cell engraftment were estimated by measuring the presence of human albumin in peripheral blood, using the human albumin ELISA quantitation kit (Bethyl Laboratories, #E80-129). To evaluate the AAV transduction potential, mice were placed on 10% NTBC and were maintained in this condition until harvest.<br>Mice were assigned to experiments and injected via intravenous route (lateral tail vein) with the indicated vector doses, and were 10 to 16 weeks of age at the point of injection.<br><br>A young adult male Macaca fascicularis NHP animal was subjected to immunoadsorption and subsequently dosed intravenously. At the moment of injection, this animal was 2 years and 11 months old.
Wild animals	Study did not include wild animals.
Reporting on sex	All the humanized FRG utilized in this study were female.<br>A young adult male non-human primate (Macaca fasicularis).
Field-collected samples	Study did not include samples collected from the field.
Ethics oversight	All murine experimental procedures and care were approved by the joint Children's Medical Research Institute (CMRI) and The Children's Hospital at Westmead Animal Care and Ethics Committee. For the non-human primate study, Animal procedures were approved by the ethical committee for animal testing of the University of Navarra and by the Department of Health of the government of Navarra (Comité de Etica para la Experimentación Animal code: 038/15) and performed according to the guidelines from the institutional ethics commission.

Note that full information on the approval of the study protocol must also be provided in the manuscript.
